# Contraceptive Method at First Sexual Intercourse and Subsequent Pregnancy Risk: Findings from a Secondary Analysis of 16-Year-Old Girls from the RIPPLE and SHARE Studies

**DOI:** 10.1016/j.jadohealth.2008.06.006

**Published:** 2009-01

**Authors:** Alison Parkes, Daniel Wight, Marion Henderson, Judith Stephenson, Vicki Strange

**Affiliations:** a Medical Research Council Social and Public Health Sciences Unit, University of Glasgow, Glasgow, United Kingdom; b UCL Centre for Sexual Health and HIV Research, Department of Primary Care and Population Sciences, University College London, London, United Kingdom; c Social Science Research Unit, Institute of Education, University of London, London, United Kingdom

**Keywords:** Oral contraception, Pregnancy, Adolescent

## Abstract

**Purpose:**

Existing failure rate studies indicate that typical use of oral contraception (OC) results in fewer unplanned pregnancies than condom use, even among teenagers. However, comparative data on pregnancy risk associated with different contraceptive methods are lacking for younger teenagers starting their first sexual relationship. This study examined associations between contraceptive method at first intercourse and subsequent pregnancy in 16-year-old girls.

**Methods:**

Six thousand three hundred forty-eight female pupils from 51 secondary schools completed a questionnaire at mean age 16 years; 2,501 girls reported sexual intercourse. Logistic regression (N = 1952) was used to model the association of contraceptive method at first intercourse with pregnancy.

**Results:**

At first intercourse (median age 15 years) 54% reported using condoms only, 11% dual OC and condoms, 4% OC only, 4% emergency contraception, and 21% no effective method. Method used was associated with a similar method at a most recent intercourse. One in 10 girls reported a pregnancy. When compared to use of condoms only, greater pregnancy risk was found with no effective method (odds ratio [OR] 2.97, 95% confidence interval [CI] 2.12–4.15) or OC only (OR 2.44, 95% CI 1.29–4.60). Pregnancy risk for dual use and emergency contraception did not differ from that for condoms only. Both significant effects were partially attenuated by adjusting for user characteristics and sexual activity.

**Conclusions:**

Young teenagers may use OC less efficiently than condoms for pregnancy prevention. The characteristics of those using OC-only confirm vulnerability to unintended pregnancy, and suggest that alternative contraceptive strategies should be considered for these young women.

Failure rates for different methods of contraception over the first year of typical use suggest that, in general, condoms offer less protection than oral contraceptives (OC): a review of U.S. studies found 15% of women using condoms experienced an unintended pregnancy in the first year of typical use, compared to 8% of those using OCs [1]. Two U.S. studies found that the unintended pregnancy rate among teenagers using condoms for 1 year was about twice that for teenagers using OC [2,3]. However, failure rate studies have not distinguished between those in their early and those in their late teens. Evidence on whether young teenage girls are more likely to avoid getting pregnant by using OC rather than condoms is currently lacking.

Differences in the protective effect of two methods measured in failure studies largely reflect differences in how effectively each method is used, rather than inherent properties of methods when used perfectly [4]. Although a large study of U.S. women found that those younger than 18 years used OC as consistently as older women [5], other research suggests that those aged 16 or younger may be less reliable [6,7]. Younger adolescents' compliance may be handicapped by the need to conceal sexual activity, by erratic routines including sporadic sexual activity and by concerns over side effects [6,8,9]. However, teenagers may also have more difficulty using condoms: misconceptions about correct use, errors in use, and failure through slippages and breakage are all relatively common among young people [10–13].

In the United Kingdom, concerns over the high level of teenage pregnancies led to the foundation of the Teenage Pregnancy Unit in 1999, with the target of halving the rate of under-18 conceptions in England, together with similar initiatives in the other countries of the United Kingdom [14]. Along with projects to improve sex education and target deprived areas, improving access to sexual health services is an important policy focus. Most U.K. teenagers rely on condoms at first sexual intercourse (FSI) (a national survey found over 80% of teenage girls aged 16–19 had used condoms at first intercourse, and 25% had used OC either with or without condoms [15]). OC may be obtained only from a medical practitioner, but patient confidentiality is protected even for the under 16s. Although falling within the general remit of sex education and counseling services, there is less focus on efficient use of OC than on correct condom use. The contribution of incorrect or inconsistent contraceptive use to teenage pregnancy is not well understood, with a lack of U.K. information on failure rates.

In this U.K.-based study of girls in their midteens, we examined pregnancy risk associated with contraceptive method at first intercourse. We used data from two randomized control trials of sex education in U.K. secondary schools (RIPPLE and SHARE), conducted with similar age groups (13–14 years RIPPLE, 13–15 years SHARE) and followed up with similar questionnaires given to equivalent school year groups (aged 15–16 years). The RIPPLE intervention consisted of a peer-led sex education program (delivering three class sessions to each year group after 3 days' peer leader training), whereas SHARE was an enhanced teacher-led sex education program (delivering 20 class sessions to each year group after 5 days' teacher training). SHARE baseline data were representative of the 1991 census Scottish population in terms of parental social class and family composition [16]. RIPPLE baseline data were representative of 1991 census English population in terms of privately owned accommodation, and of 1998 GCSE education qualifications [17].

Our main research question was whether teenagers using OC only were any more, or any less, protected against pregnancy than those using condoms only. We have included girls who reported not using a reliable form of contraception as well as other contraception groups for comparison purposes. An analysis of the SHARE data set has already shown [18] that nonuse of contraception at first intercourse is associated with pregnancy in 15- to 16-year-old girls, echoing similar U.S. findings [19].

To interpret our findings, we explore whether younger age at first intercourse or other user characteristics help to account for associations between contraceptive method and pregnancy. We also consider whether social acceptability may have influenced reporting of method, and the extent to which method at first intercourse is related to subsequent contraceptive use.

## Methods

### RIPPLE dataset

Twenty-seven schools participated in the RIPPLE randomized control trial (RCT) of peer-led school sex education in England during 1997 to 2001 [17]. This trial was approved by the committee on the ethics of human research at University College London. All pupils in a year group (N = 9,508) were eligible to participate, with 8,766 pupils including 4,248 girls recruited at baseline (mean age 13 years 8 months). Pupils in 26 schools were followed up with a self-complete questionnaire at mean age 16 years 0 months (N = 6,656 including 3,230 girls).

### SHARE dataset

Twenty-five schools participated during 1996 to 1999 in the SHARE RCT of enhanced teacher-led sex education in Scotland [16]. This trial was approved by Glasgow University's Ethical Committee for Nonclinical Research Involving Human Subjects. All pupils in a year-group were invited to take part (N = 8,430). At baseline (N = 7,616 including 3,794 girls, mean age 14 years, 2 months) and follow-up (N = 5,854 including 3,118 girls, mean age 16 years, 1 month) pupils provided information in a self-complete questionnaire.

### Eligibility

To be included in the analysis of association between contraceptive method reported at FSI and pregnancy, female subjects responding to the questionnaire at age 16 follow-up had to meet the following eligibility criteria: (1) have reported sexual intercourse in the questionnaire, and (2) have responded to the full version of the questionnaire. The second criterion was important only in the SHARE study. Nine SHARE schools in one education authority were not asked questions about sensitive aspects of sexual experience, including pregnancy. In addition, SHARE early school leavers who did not answer the full postal questionnaire were then sent a shorter version that omitted questions on contraception at FSI.

### Main measures

The main outcome measure was the “yes” response to the question asked of girls in both studies: “Have you ever been pregnant?”

Girls in both studies were asked about contraception at FSI (“When you first had sexual intercourse, did you or your partner use any form of contraception or do anything to protect yourselves?” and invited to tick all of the options that applied to them from the following list: none, withdrawal, condom, OC, emergency contraception, “don't know” and “other” (with a request to write in what was used in this last category). Responses were recoded into five groups: (1) condom only, (2) dual protection (OC with condom), (3) OC only, (4) emergency contraception only, (5) “no effective method,” defined as no use of barrier or hormonal contraception. A sixth category was created for don't know/missing response.

### Analysis

Most of the analysis combined girls from both arms of each RCT study. In doing so we took the precaution of adjusting for study group, arm of trial and a term that allowed for an interaction between the two factors. Neither study had found differences between intervention and control arms in contraceptive behavior. The RIPPLE study found a borderline effect of lower unintended pregnancy among girls in the intervention arm reported at age 16 (2.3% vs. 3.3%, *p* = 0.07), although there was no corresponding between-arm difference in the SHARE study [16,17]. Combining the data sets allowed for increased statistical power to detect differences in the likelihood of pregnancy for contraceptive groups with small numbers of individuals in each data set.

There were three stages to a logistic regression modeling the association between contraceptive method at first intercourse and pregnancy. The first stage adjusted for study and trial arm as described, together with age at follow-up in months. The logistic regression was performed using MLwiN v. 2.0, which took account of clustering by school and weighted cases to counteract the effects of differential attrition from baseline to follow-up. For SHARE, the main variables contributing to the weights were early school leaver status, gender, social class, family composition, parental monitoring, spending money, and drunkenness. In RIPPLE, the main variables contributing to the weights were school randomization stratum and randomization arm, housing tenure, smoking habits at baseline, attitude toward skipping school, religion, expectation of being a parent by age 20, having had sex by intermediate follow-up, and knowledge of contraceptive services.

The second stage contained an adjustment for age at FSI (combining all those aged 11 years or younger). The third stage in the logistic regression did not adjust for age at FSI, but contained adjustments for a fuller set of measures related to deprivation, expectations, and sexual lifestyle. These measures were selected from a list of factors previously identified as being associated with teenage pregnancy [20], if they were found to have significant (*p* < .05) bivariate associations with both pregnancy and OC-only use in our combined dataset.

In all stages, dummies were included for missing values of independent variables. Interactions between study group and other independent variables were tested and reported on where significant (*p* < .05).

Following this analysis, we explored whether declared OC-only usage was any more likely than condom use to reflect an element of socially acceptable reporting. To this end, we investigated whether circumstances known to be strongly associated with lack of protection (such as partner pressure to have intercourse, or lack of planning) were more likely to be associated with OC-only reporting.

We also considered possible reasons why girls may have preferred OC to condoms. Here we compared baseline differences in knowledge of STIs and attitudes to condoms between OC-only and condom use groups.

Finally, we examined whether contraceptive method at FSI was associated with contraceptive method at most recent intercourse.

## Results

There were 2,082 girls from both datasets who were eligible for this study. Multivariate analyses used a subset of the total eligible sample with complete information on the pregnancy measure (N = 1,952). Table 1 provides further details on sample selection and characteristics. The SHARE sample contained a lower proportion of girls from ethnic minority groups, and a higher proportion from single/no parent families and deprived social backgrounds than the RIPPLE sample (all *p* < .001).

The age distribution for FSI was skewed, with 17% under the age of 14 years and 54% aged 15 or 16 years. The median age for FSI was 15 years. Most girls (65%) reported using a condom at FSI (Table 2, column 1). Fifteen percent reported using OC, although only 4% used OC without a condom. One in four did not report an effective method of contraception at intercourse, although approximately one in five of this group used emergency contraception after sex. None reported use of long-lasting hormonal methods such as injectables or implants. Girls from the SHARE study were more likely to report no effective method, and less likely to have missing contraceptive information than those in RIPPLE. Use of other methods was similar in the two studies. Almost one in 10 girls (N = 163) reported a pregnancy by the follow-up survey, with similar rates in the two studies.

There were no significant (*p* < .05) effects on pregnancy associated with age at follow-up, arm of trial or study group at any stage of modelling. Table 2 presents pregnancy odds associated with contraceptive method, the reference group being those using condoms only. In Stage 1 (first column of ORs) there was no significant difference in the likelihood of pregnancy associated with dual use of condoms and OC, with emergency contraception or with missing contraceptive information. No effective method of contraception and OC-only were both significantly associated with over twice the risk of pregnancy. The sizes of the effects were similar in both data sets, although OC-only bordered on significance in the SHARE data set. There was no interaction effect between study group and contraceptive method. Stage 1 *R*^2^ was 0.07 (compared to .01 in the null model without contraceptive method).

In Stage 2, adjusting for age at FSI attenuated the effect for nonuse of contraception but slightly increased the odds related to OC-only use in the combined and separate datasets (Table 2, second column of ORs).

The social background, expectations, risk behaviours and sexual lifestyle of girls reporting OC-only use at FSI were compared with those reporting condoms (with or without OC) using a subset of those reporting sexual experience at follow-up, who had responded to both the baseline and full follow-up questionnaires (N = 2,091, with a subset N = 1,590 reporting condom and/or OC use). Table 3 presents odds ratios that are adjusted for study and arm of trial, but are not adjusted for other factors listed in the table. OC-only users were more likely to come from deprived family backgrounds, to expect early parenthood and to have more sexual partners. The effects of deprivation and expectations on pill use were similar in the two studies, but the effect of number of partners was more pronounced in the SHARE study (not shown). Deprivation, expectations of early parenthood and number of sexual partners were also independently associated with pregnancy (not shown). There was a significant interaction between study and deprivation, where deprivation had a greater effect on pregnancy in the SHARE study.

The third stage of the logistic regression adjusted for deprivation, expectations, and number of partners (together with the interaction term for study group and deprivation in the combined dataset): see last column of Table 2. As in Stage 2, there was a downward adjustment of the odds related to no effective contraceptive method in both datasets. Overall, there was also a decrease in the odds relating to OC-only use, although this was attributable to effects in the SHARE dataset only. In both Stages 2 and 3 there was a substantial increase in *R*^2^ compared to Stage 1.

Were girls more likely to report OC than condoms for socially acceptable reasons? On the whole, girls were no more likely to report OC-only use in circumstances known to be associated with a lack of effective contraception than they were to report using condoms (Table 4). The main exception was that OC-only was less likely to involve partner communication about contraception than condom use.

Table 4 also suggests reasons why girls may have preferred OC to condoms. OC-only users were more likely to find condoms difficult to use and embarrassing. They were less likely to be aware that not all STIs can be cured with current medical treatment, although equally likely to know that not all STIs produce symptoms.

Finally, we present data showing associations between contraceptive method at FSI and contraception at most recent intercourse (Table 5). Method continuation was strong for all groups except for those using emergency contraception at FSI (Cramer's V *p* < .001). For SHARE only, method continuation was supported by information from two further separate questions on condom and OC use over respondents' entire sexual history. In the combined data set, those using OC-only were no more likely to report nonuse of contraception at most recent intercourse than those who had used condoms at FSI (chi-square NS). However, those reporting emergency contraception were more likely than condom users to be unprotected at most recent intercourse (chi-square *p* < .001).

## Discussion

The study found that teenage girls aged 16 years or under who reported OC-only use at first intercourse were more likely to become pregnant than those reporting condoms only. The study also confirmed other findings that nonuse of contraception at FSI is associated with subsequent pregnancy [18]. However, there was no difference in pregnancy risk according to whether condoms were used alone or with OC. Contraceptive behavior measured at two time points in the combined dataset (first and most recent intercourse) suggested that the risk of using OC-only compared to condoms is likely to reflect continued ineffective use of OC-only, rather than greater risk of discontinuation. It is surprising to find no difference in pregnancy risk between emergency contraception and condom use, because we found greater risk of discontinuation of contraception associated with emergency contraception.

The explanatory power of contraceptive method at first intercourse in the pregnancy models was limited, and more information on typical OC-only use in young adolescent girls over a period of time is desirable to provide firmer evidence on associations between method and pregnancy. This could be obtained from diary studies and other methods including microelectronic pill packs or biological markers [4,9]. Comparable studies are also required for other methods: as research has already indicated, adolescent condom use may vary with relationship quality, duration, and coital frequency [21,22].

In the combined data set, girls' OC-only use increased from a small group at first intercourse (4%) to almost one in five teenagers (19%) at most recent intercourse. A weakness of the study is that we cannot use OC-only use at most recent intercourse to predict pregnancy, as we do not know whether girls fell pregnant before or after this event. OC-only use at most recent intercourse was also associated with increased likelihood of pregnancy (data not shown), but this could in part be because of reverse causation, if girls switched to OC as a more reliable contraceptive method *following* a pregnancy caused, for example, by condom failure.

Other potential limitations of the study include the use of combined datasets from RCTs and a risk of bias. Although neither intervention had a significant effect on contraceptive behavior or pregnancy we routinely adjusted for arm of trial in all models, and adjusted for study group plus an interaction term for study and arm of trial when modeling the combined datasets (all effects NS). Both studies experienced differential attrition from baseline to follow-up, but weights were used to restore the representative nature of both study samples. The net effect of listwise deletion of two SHARE groups is likely to have been small. Most of the excluded SHARE pupils (from one education authority with missing pregnancy information) were more likely to report use of effective contraception, but their OC-only use did not differ from the rest. The smaller group, excluded because they only completed a short postal questionnaire, were less likely to report use of effective contraception and more likely to report OC-only at most recent intercourse.

The validity of sensitive self-reported information is another important limitation to this study. Our data do not provide any evidence to support the notion that there was a greater element of social acceptability in reports of OC-only use than in reports of condom use. Girls were no more likely to report OC use than condom use in circumstances known to contribute to lack of any protection [23]. Girls using OC only were less likely to talk with their partners about contraception before intercourse than condom users, but this is not surprising given the difference in need for partner involvement.

Oral contraception is only available in the United Kingdom after medical consultation, and teenagers in both studies were asked about use of sexual health services (including general practitioners). It is not possible to use reports of visit to sexual health services as a clear-cut check of OC reporting, as we do not know whether service visits were made before or after FSI. Sixteen girls reporting OC-only use did not report use of sexual health services: 13 were from the RIPPLE study where the questionnaire asked about use of services “for advice or information on sex” rather than “obtaining contraception” (specified as a reason for service use in the SHARE questionnaire). Eliminating girls who did not report service use did not affect the findings: the pregnancy odds for the OC-only group is revised to 2.82 (95% CI 1.42, 5.50).

We are left with the interpretation that girls were not using the pill effectively. Why might this have been the case? Within our sample, younger age at FSI did not help to explain the risk associated with OC-only use. Adjusting for factors likely to relate to greater ambivalence about pregnancy risk and increased sexual activity went some way toward explaining associations between OC-only use and pregnancy. These effects were confined to the SHARE dataset, where there were higher levels of deprivation and stronger associations between deprivation and pregnancy. Higher contraceptive failure rates are found among lower income women and those seeking to delay rather than prevent pregnancy [3,24], and other research has linked both ambivalence and more partners with inconsistent contraceptive use [25,26].

We do not know whether ineffective OC use reflected dislike of side effects, difficulties over concealment, or chaotic sexual lifestyle as suggested by previous studies of adolescent OC compliance [6,8,9]. Poor understanding of oral contraception may also have contributed to risk miscalculation [27,28]. Some girls may have been prescribed the pill for menstrual problems, and may not have acquired sufficient knowledge to use it correctly as a contraceptive.

This study suggests that for young teenagers in their first sexual relationship, condoms may be easier than oral contraception to use effectively: reinforcing existing advice that condoms should be used for sexually transmitted infection (STI) protection. Our data indicated that OC-only users were more likely to find condoms embarrassing and less easy to use, and they had poorer knowledge of STIs. Health professionals have scope to improve teenagers' attitudes toward condoms [29]. However, dual protection remains relatively uncommon among teenagers [30], and our study did not suggest that it lowered pregnancy risk compared to those reporting condoms only.

At the time of the study, long-acting reversible hormonal methods were not widely available in the United Kingdom, but they may have a useful role in pregnancy prevention without increasing STI risk [31]. Current uptake remains low among U.K. teenagers: barriers to use include side effects such as irregular bleeding and low practitioner skills in prescribing [32]. A recent study found a significant increase in OC prescription in Scotland to teenagers under 16 years [33], yet use of the oral contraceptive pill by girls who start sex under 16 should not be regarded as a simple or sufficient approach to tackling teenage pregnancy. Our findings support the need for counseling to ensure proper understanding of common side effects, good compliance, and what to do when a pill is missed [34], although to date it has proved difficult to devise ways to improve adherence to an OC regime [35].

## Figures and Tables

**Table 1:** Girls in the RIPPLE and SHARE datasets: sample selection and characteristics

	RIPPLE N	SHARE N	Combined N
Responded at baseline	4,248	3,794	8,042
Responded at age 16 follow-up	3,230	3,118	6,348
Reported intercourse at age 16 follow-up	1,222	1,279	2,501
Excluded: not asked questions on pregnancy		−368	
Excluded: not asked questions on contraception at FSI		−51	
Eligible sample	1,222	860	2,082
Modeling sample (complete information on pregnancy)	1,109	843	1,952

Composition of modelling sample (weighted percentages)	%	%	%
Ethnic group			
White	90	98	93
Black	1	0	1
Asian	0	0	0
Other	9	2	6
Family structure			
Live with both biological parents	62	57	60
Live with one or neither biological parent	38	43	40
Deprivation score^a^			
Zero	28	18	24
One	31	27	30
Two	26	31	28
Three or four	15	23	18

FSI = first sexual intercourse.

a Deprivation: count of social housing, father left school at 16, mother left school at 16, neither parent in FT employment.

**Table 2:** Association of contraceptive method at first intercourse with pregnancy reported at age 16 follow-up, RIPPLE and SHARE data sets

	N (column %) in contraception group	N (row %) pregnant	Stage 1		Stage 2 (1 + adjustment for agent FS)		Stage 3 (1 + adjustment for deprivation, expectations and number of partners)	
			OR	95% CI	OR	95% CI	OR	95% CI
All girls								
Total	1,952 (100)	163 (10)						
Condom only	1,109 (54)	67 (7)	1.00		1.00		1.00	
Dual (condom and OC)	198 (11)	9 (6)	0.82	(0.45–1.52)	0.80	(0.43–1.51)⁎	0.80	(0.43–1.48)⁎
OC only	73 (4)	11 (16)	2.44	(1.29–4.60)⁎	2.49	(1.27–4.88)⁎	2.17	(1.12–4.19)⁎
Emergency contraception	72 (4)	4 (5)	0.67	(0.23–1.94)	0.66	(0.22–1.93)	0.67	(0.23–1.98)
No effective method	380 (21)	61 (18)	2.97	(2.12–4.15)⁎	2.61	(1.83–3.71)⁎	2.56	(1.81–3.63)⁎
Missing	120 (6)	11 (10)	1.61	(0.86–3.02)	0.66	(0.22–1.97)	1.50	(0.79–2.83)
*R*^2^ (null model = .01)			0.07		0.26		0.12	
RIPPLE girls								
Total	1,109 (100)	86 (9)						
Condom only	617 (54)	34 (6)	1.00		1.00		1.00	
Dual (condom and OC)	132 (12)	7 (6)	1.06	(0.50–2.23)	0.97	(0.45–2.10)	1.11	(0.52–2.36)
OC only	39 (4)	5 (15)	2.97	(1.23–7.14)⁎	3.15	(1.26–7.90)⁎	3.17	(1.28–7.85)⁎
Emergency contraception	33 (3)	3 (8)	1.32	(0.37–4.66)	1.04	(0.28–3.87)	1.45	(0.41–5.22)
No effective method	179 (17)	28 (18)	3.44	(2.11–5.60)⁎	3.26	(1.94–5.47)⁎	2.93	(1.76–4.87)⁎
Missing	109 (10)	9 (10)	1.70	(0.85–3.39)	2.05	(1.00–4.20)	1.68	(0.83–3.41)
*R*^2^ (null model = .01)			0.07		0.26		0.13	
SHARE girls								
Total	843 (100)	77 (11)						
Condom only	492 (55)	33 (8)	1.00		1.00			
Dual (condom and OC)	66 (8)	2 (5)	0.54	(0.18–1.64)	0.63	(0.20–2.00)	0.50	(0.16–1.53)
OC only	34 (4)	6 (16)	2.33	(0.92–5.85) *p* < .1	2.64	(0.96–7.25) *p* < .1	1.86	(0.71–4.89)
Emergency contraception	39 (4)	1 (3)	0.25	(0.03–2.17)	0.30	(0.03–2.61)	0.24	(0.03–2.11)
No effective method	201 (27)	33 (19)	2.56	(1.62–4.05)⁎	2.16	(1.33–3.51)⁎	2.16	(1.34–3.47)⁎
Missing	11 (1)	2 (18)	2.10	(0.41–10.74)	1.41	(0.26–7.70)	1.71	(0.30–9.65)
*R*^2^ (null model = .01)			0.09		0.29		0.17	

OR = odds ratio; CI = confidence interval; OC = oral contraceptive.

⁎ Denotes *p* < .05. All models adjusted for arm of trial and age in months at follow-up. Combined model also adjusted for study group and interaction term (study × arm of trial).

**Table 3:** Association of social background, expectations, risk behaviors, and sexual lifestyle with OC-only use rather than condoms at first intercourse, reported by girls in combined data set

N = 1,590 (all girls reporting condom and/or OC use)	N (column %) in group	N (row %) using OC-only	OR	95% CI
Baseline measures reported at age 13/14				
Deprivation score^a^				
none	421 (25)	12 (3)	1.00	
1	490 (30)	22 (5)	1.76	(0.87–3.58)
2	438 (28)	34 (8)	2.99	(1.66–5.37)⁎
3 or 4	241 (17)	17 (8)	2.88	(1.29–6.42)⁎
Like school				
Agree	1,289 (80)	62 (5)	1.00	
Don't agree	295 (20)	23 (8)	1.55	(0.87–2.75)
Regular use of cigarettes, alcohol or cannabis^b^				
no	1,149 (71)	57 (5)	1.00	
yes	386 (29)	26 (8)	1.54	(0.88–2.71)
Expect early parenthood^c^				
no	1,350 (84)	64 (5)	1.00	
yes	217 (16)	20 (11)	2.32	(1.42–3.80)⁎
Expect attend college/university^c^				
yes	1,038 (64)	46 (5)	1.00	
no	538 (36)	39 (8)	1.54	(0.98–2.42)
Sexual behavior reported at age 16 follow-up				
Frequency of intercourse in last 12 months				
less than 10 times	1,085 (66)	39 (5)	1.00	
10+ times	505 (32)	44 (11)	1.45	(0.95–2.22)
Number of sexual partners in last 12 months				
less than 3	1,363 (86)	57 (6)	1.00	
3 or more	202 (14)	25 (16)	2.08	(1.26–3.42)⁎

OR = odds ratio; CI = Confidence interval. ORs are adjusted for study and arm of trial but not adjusted for other measures in the table.

a Deprivation: count of social housing, father left school at 16, mother left school at 16, neither parent in FT employment.

b Regular cigarette and cannabis use were highest points on four-point usage scale (“never”, “tried”, “occasional”, and “regular”), regular alcohol was drunkenness once a week or more, representing two highest points on five-point scale (“never”, “once a year”, “once a month”, “once a week”, “more than once a week”).

c Expectations were converted to binary from five-point scale (“very likely” to “very unlikely”) of responses to questions asked about expectations for age 18 (SHARE) or age 20 (RIPPLE).

⁎ Denotes *p* < .05.

**Table 4:** Contextual and attitudinal factors associated with OC-only use rather than condoms at first intercourse, reported by girls in combined data set

N = 1,590 (all girls reporting condom and/or OC use)	N (column %) in group	N (row %) using OC-only	OR	95% CI
Aspects of first intercourse reported at follow-up associated with no contraception in previous studies				
Age at first intercourse				
14 years or older	850 (81)	45 (6)	1.00	
Under 14 years	179 (19)	13 (8)	1.33	(0.74–2.38)
Relationship with partner prior to intercourse				
More than 1 month	942 (60)	46 (5)	1.00	
1 month or less	629 (40)	37 (6)	1.28	(0.84–1.95)
Pressure from partner				
No	1,332 (84)	72 (6)	1.00	
Yes	247 (16)	11 (5)	0.80	(0.43–1.48)
Planning				
Some anticipation	981 (65)	51 (6)	1.00	
Unplanned	514 (35)	28 (6)	1.01	(0.57–1.79)
Drunk/stoned				
No	1,206 (77)	67 (6)	1.00	
Yes	371 (23)	16 (5)	0.68	(0.37–1.23)
Talked about contraception with partner before intercourse				
Yes	1,080 (74)	49 (5)	1.00	
No	390 (26)	31 (9)	1.81	(1.13–2.91)⁎
Baseline measures relating to preference for OC over condoms				
Condom self-efficacy^a^				
High	764 (50)	33 (5)	1.00	
Medium	531 (33)	29 (6)	1.34	(0.85–2.12)
Low	271 (16)	21 (9)	2.20	(1.27–3.80)⁎
Condoms embarrassing to use				
Don't agree	1,437 (93)	69 (5)	1.00	
Agree	102 (7)	14 (18)	4.25	(2.32–7.79)⁎
Knowledge (1): STIs do not all have symptoms				
Correct answer	1,004 (64)	53 (6)	1.00	
Incorrect answer	555 (36)	29 (6)	1.17	(0.75–1.81)
Knowledge (2): STIs cannot all be cured with medical treatment				
Correct answer	1,265 (80)	57 (5)	1.00	
Incorrect answer	288 (20)	25 (9)	1.90	(1.18–3.04)⁎

OR = odds ratio; CI = confidence interval; OC = oral contraceptive; STI = sexually transmitted infection. ORs are adjusted for study and arm of trial but not adjusted for other measures in the table.

a Condom self-efficacy: tertiles of mean scores from questions on how easy to get condoms, to suggest use to partner and to use them properly, answers coded on five-point scale from “strongly agree” to “strongly disagree.”

⁎ denotes *p* < .05.

**Table 5:** Association of contraceptive method at FSI with contraception at most recent intercourse, reported by girls in combined data set

N = 1,493	Contraceptive method at FSI				
	Condom only %	Dual (condom and OC) %	OC-only %	Emergency contraception %	No effective method %
Contraceptive method at most recent intercourse					
Condom only	57	11	6	33	20
Dual (condom and OC)	11	61	13	6	7
OC-only	17	20	61	20	18
Emergency contraception	2	2	9	11	2
No effective method	13	7	11	30	53
All	100	100	100	100	100

OC = oral contraceptive; FSI = first sexual intercourse. Analysis excludes those with missing information on contraceptive methods, %s are weighted values.
